# From the ‘black box' to ‘domino effect' mechanism: what have we learned from the structures of respiratory complex I

**DOI:** 10.1042/BCJ20210285

**Published:** 2023-03-15

**Authors:** Leonid A. Sazanov

**Affiliations:** Institute of Science and Technology Austria, Am Campus 1, Klosterneuburg 3400, Austria

**Keywords:** complex I, electron transfer, membrane protein structure, proton pumping, respiratory chain

## Abstract

My group and myself have studied respiratory complex I for almost 30 years, starting in 1994 when it was known as a L-shaped giant ‘black box' of bioenergetics. First breakthrough was the X-ray structure of the peripheral arm, followed by structures of the membrane arm and finally the entire complex from *Thermus thermophilus*. The developments in cryo-EM technology allowed us to solve the first complete structure of the twice larger, ∼1 MDa mammalian enzyme in 2016. However, the mechanism coupling, over large distances, the transfer of two electrons to pumping of four protons across the membrane remained an enigma. Recently we have solved high-resolution structures of mammalian and bacterial complex I under a range of redox conditions, including catalytic turnover. This allowed us to propose a robust and universal mechanism for complex I and related protein families. Redox reactions initially drive conformational changes around the quinone cavity and a long-distance transfer of substrate protons. These set up a stage for a series of electrostatically driven proton transfers along the membrane arm (‘domino effect'), eventually resulting in proton expulsion from the distal antiporter-like subunit. The mechanism radically differs from previous suggestions, however, it naturally explains all the unusual structural features of complex I. In this review I discuss the state of knowledge on complex I, including the current most controversial issues.

## Introduction

In the respiratory chain, oxidative phosphorylation (OXPHOS) enzymes are responsible for energy production (in the form of ATP) in eukaryotic cells and in many bacteria [1]. In mitochondria the chain consists of four large inner membrane-embedded protein complexes — complex I (CI) or NADH-ubiquinone oxidoreductase, complex II (CII) or succinate dehydrogenase, complex III_2_ (CIII_2_) or cytochrome *bc*_1_ oxidoreductase and complex IV (CIV) or cytochrome *c* oxidase [2]. The proton motive force (*pmf*) generated by the concerted action of these enzymes drives ATP synthase [3]. Over the years we have worked on most respiratory enzymes, studying them either on their own or as part of supercomplexes, in which they are often organised *in situ* [4]. Our aim is to understand the basic mechanisms of these huge molecular machines, especially complex I. In the course of the 1990s, as the structures of the respiratory enzymes were first determined, it became clear that they all operate on very different principles of coupling between redox reactions and proton translocation – Q-cycle for CIII_2_ [5], direct coupling for CIV [6] and rotary for ATPase [7]. However, complex I, being the largest and most elaborate (comprising up to 45 subunits of ∼1 MDa MW in total in mammals) assembly of the chain, remained the ‘block box' for much longer. All that was known about complex I structure was that it has an L-shape formed by the peripheral and membrane arms [8].

We first studied bovine complex I by 2D electron crystallography [9], which was one of the main methods to study membrane proteins earlier but is rarely used nowadays, surpassed by the new single particle EM approaches. Using a simpler bacterial complex I (14 conserved subunits) as a model, we could show that antiporter-like subunits ND5/NuoL and ND4/NuoM (bovine/*E. coli* nomenclature) form a distal part of the membrane arm, with limited information otherwise due to low resolution [10,11]. At that time we decided to switch to complex I from *Thermus thermophilus*, as likely more stable enzyme, even though it was never purified before. This has lead to a first major breakthrough in 2006 — structure of the peripheral arm determined by X-ray crystallography [12]. Our extensive efforts on the crystallisation of *E. coli* enzyme eventually lead to the X-ray structure of the membrane arm [13]. Crystals of the entire *E. coli* enzyme were obtained but never diffracted to high resolution, however that was fortunately not the case for *T. thermophilus.* Although *T. thermophilus* crystals were always twinned, severely complicating structure solution*,* in 2013 we finally were able to solve the first structure of the entire complex I [14]. Shortly after that the cryo-EM ‘resolution evolution' happened, prompting us to apply these new approaches to mammalian complex I, resulting in the first nearly complete (88% atomic) structure of the mammalian (ovine) complex I [15]. It should be noted that simultaneously published structure of the bovine enzyme [16] was only ∼53% atomic due to lower resolution (i.e. 47% of residues had no side chains).

Thus, by 2016 we knew the structures of bacterial and mammalian complex I, allowing for a range of hypotheses on the coupling mechanism to be developed in the subsequent years. These were mostly based on the long-range conformational changes due to a large distance (up to 200 Å) between electron transfer reactions (happening in the peripheral arm) and proton translocation across the membrane arm [17–24]. The proposals varied in many details in the absence of hard experimental evidence (high-resolution structures with resolved waters) on what happens with enzyme during turnover. Recently we succeeded in resolving such structures first for mammalian [25] and then for bacterial [26] enzyme, allowing us to develop a detailed, robust and universal coupling mechanism of complex I. It turned out to be radically different from previous models, leading to intense discussions but also some emerging consensus, as presented below.

## Basics of complex I architecture

**Figure 1.**
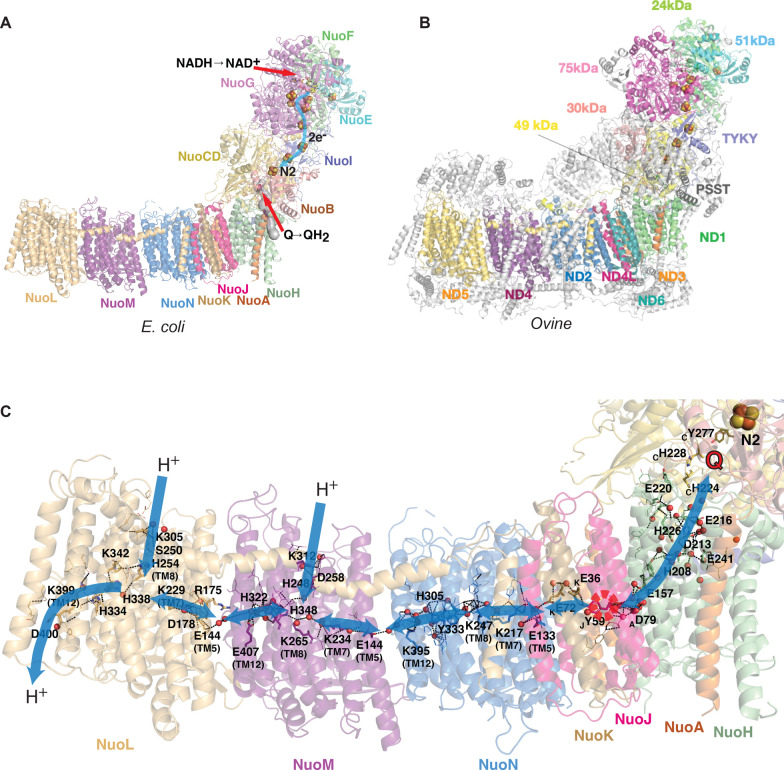
Architecture of complex I. (**A**) Structure of the bacterial (*E. coli*) complex I [26] with subunits coloured and annotated. Substrates and cofactors are indicated. Electron transfer path is indicated by light blue arrow. Quinone cavity is shown as grey surface. (**B**) Structure of the mammalian (ovine) complex I [25] with 14 core subunits coloured and annotated and the remaining supernumerary subunits shown in grey. (**C**) Proton transfer pathways, outlined by blue arrows. Membrane arm contains the central axis of charged residues, essential for the proton transfer and the coupling. Structure of the *E. coli* open state [26] is shown coloured by subunit, with essential residues shown as sticks. Key ALS residues are also identified by their TM helix. Experimentally observed waters are shown as red spheres (waters beyond 5 Å from essential residues are omitted for clarity). Putative proton pathways through Grotthus-competent residues (shown as lines unless key residue) and waters are shown as black dashes. The break at NuoJ ND3 π-bulge is indicated by the red dashed circle.

The general architecture of complex I is conserved, comprising fourteen core subunits (in bacteria) equally divided between the peripheral arm (PA) and the membrane arm (MA) (Figure 1A). Eukaryotes contain additional supernumerary (or accessory) subunits, which form a shell around the core (Figure 1B). These (mammalian enzyme has 31 such subunits) are likely not involved in the catalytic reaction but assist with the assembly, stability and regulation of the complex I [15,27].

The seven conserved core subunits of the PA can be assigned to the N- (NADH-binding) and Q- (quinone-binding) modules (Table 1). Here, electrons from NADH are accepted (as a hydride) by flavin-mononucleotide (FMN) and then passed by electron tunnelling along a chain of seven iron-sulfur clusters (ending with cluster N2) to the final acceptor quinone (Figure 1A) [26,28]. Additional cluster N1a lies off-path ‘upstream' of FMN, and its role could be to temporarily store an electron to prevent flavosemiquinone formation and reduce ROS generation [29,30]. The ninth FeS cluster N7, far off the main path, exists only in some bacteria and is probably an evolutionary vestige [31]. Since the largest drop in redox potential along the chain occurs between cluster N2 and the quinone/quinol pair, the crucial energy-releasing step in the reaction is therefore likely to be quinone reduction/protonation [17] or perhaps even its release out of the binding cavity as the potential of the Q/QH_2_ pair bound near N2 is likely similar to the N2 potential [19]. This has been confirmed by real-time measurements of electron transfer reactions in the peripheral arm, which also ruled out the involvement of a long-lived semiquinone radical in the mechanism [32]. For each NADH oxidised and quinol produced, four protons are pumped from the matrix to the inter-membrane space (IMS) [33].

**Table 1:** Core subunits of complex I

Module	*Escherichia coli*	*Thermus thermophilus*	*Yarrowia lipolytica*	*Bos taurus* (Bovine)	*Homo sapiens*	
Peripheral arm						Cofactors^1^
Dehydrogenase (N)	NuoF	Nqo1	NUBM	51 kDa	NDUFV1	FMN N3 (4Fe[51])
	NuoE	Nqo2	NUHM	24 kDa	NDUFV2	N1a (2Fe[24])
	NuoG	Nqo3	NUAM	75 kDa	NDUFS1	N1b (2Fe[75]), N4 (4Fe[75]C), N5 (4Fe[75]H), (N7)^2^
Connecting (Q)	NuoD (NuoCD)^3^	Nqo4	NUCM	49 kDa	NDUFS2	No cofactor
	NuoC^3^	Nqo5	NUGM	30 kDa	NDUFS3	No cofactor
	NuoI	Nqo9	NUIM	TYKY	NDUFS8	N6a (4Fe[TY]1), N6b (4Fe[TY]2)
	NuoB	Nqo6	NUKM	PSST	NDUFS7	N2 (4Fe[PS])
Membrane arm						TMH^4^
-	NuoH	Nqo8	NU1M	ND1	ND1	8–9
Pumping (P)	NuoA	Nqo7	NU3M	ND3	ND3	3
	NuoJ	Nqo10	NU6M	ND6	ND6	5
	NuoK	Nqo11	NULM	ND4L	ND4L	3
	NuoN	Nqo14	NU2M	ND2	ND2	11–14
	NuoM	Nqo13	NU4M	ND4	ND4	14
	NuoL	Nqo12	NU5M	ND5	ND5	16–17

1 The traditional nomenclature for Fe-S clusters (Nx, derived from initially described electron paramagnetic resonance (EPR) signatures [59], as well as the nomenclature proposed [60] on the basis of re-assignment of EPR signals to structurally observed clusters, is shown. In the new nomenclature, clusters are named according to their nuclearity (2Fe or 4Fe), their subunit location (using bovine nomenclature) and when necessary, as ligated by four Cys (C) or three Cys and one His (H);

2 Cluster N7 is present only in some bacteria (for example, *E. coli* and *T. thermophilus;*)

3 Subunits NuoC and NuoD are fused in *E. coli* and some other bacteri;a

4 Number of transmembrane helices.

In the MA, ND1/NuoH subunit forms part of the PA–MA interface (Figure 1A,B). It is followed by three small subunits, ND3/NuoA, ND6/NuoJ and ND4L/NuoK, which form, together with part of ND1, the E-channel containing key glutamates [14]. The rest of MA is formed by three homologous antiporter-like subunits (ALS), ND2/NuoN, ND4/NuoM and ND5/NuoL, containing 14 conserved transmembrane helices (TM) each. A central axis of charged residues connects these subunits, suggesting a way of long-distance communication along the MA (Figure 1C) [14,15,26].

ALS share a cation/proton antiporter MRP fold [34] of two inverted symmetric five TM repeats forming one half of the putative proton translocating channel each, exposed either to the matrix/cytosol (N-terminal repeat) or to the IMS/periplasm (C-terminal repeat). Both halves contain a key conserved lysine LysTM7/12 (or GluTM12 in ND4), connected in each ALS by a central LysTM8 (or HisTM8 in ND5) into apparently a full channel across the membrane. In each ALS, LysTM7 forms an ion pair with a conserved GluTM5. All of these key residues sit on breaks in TM helices (loops in TM7/TM12, π-bulge in TM8), which may render the central hydrophilic axis flexible. This has led to various mechanistic proposals involving long-range conformational changes. We suggested [14,21] that quinone reactions could ‘shift' the central MA axis so that half-channels are exposed either to the matrix or to IMS, resulting in pumping of one proton per ALS and an additional one via E-channel, matching the experimental value [33]. Alternative ‘electrostatic wave' mechanism involved co-ordinated forward and backward waves of conformational changes and charge interactions from quinone site to the tip of ND5 subunit [20,35]. An earlier version of a similar model was based on mutagenesis studies of the conserved residues of the central axis [18]. A ‘two-stroke' model suggested that two pump modules are driven in two conformational strokes generated by stabilisation of the anionic forms of semiquinone and ubiquinol [36]. While some elements of the earlier models, such as the critical role of key lysines, have stood the test of time, others turned out to be incompatible with the recent cryo-EM results [25,26,37].

## Open and closed states of complex I: a relationship to the catalytic cycle

**Figure 2.**
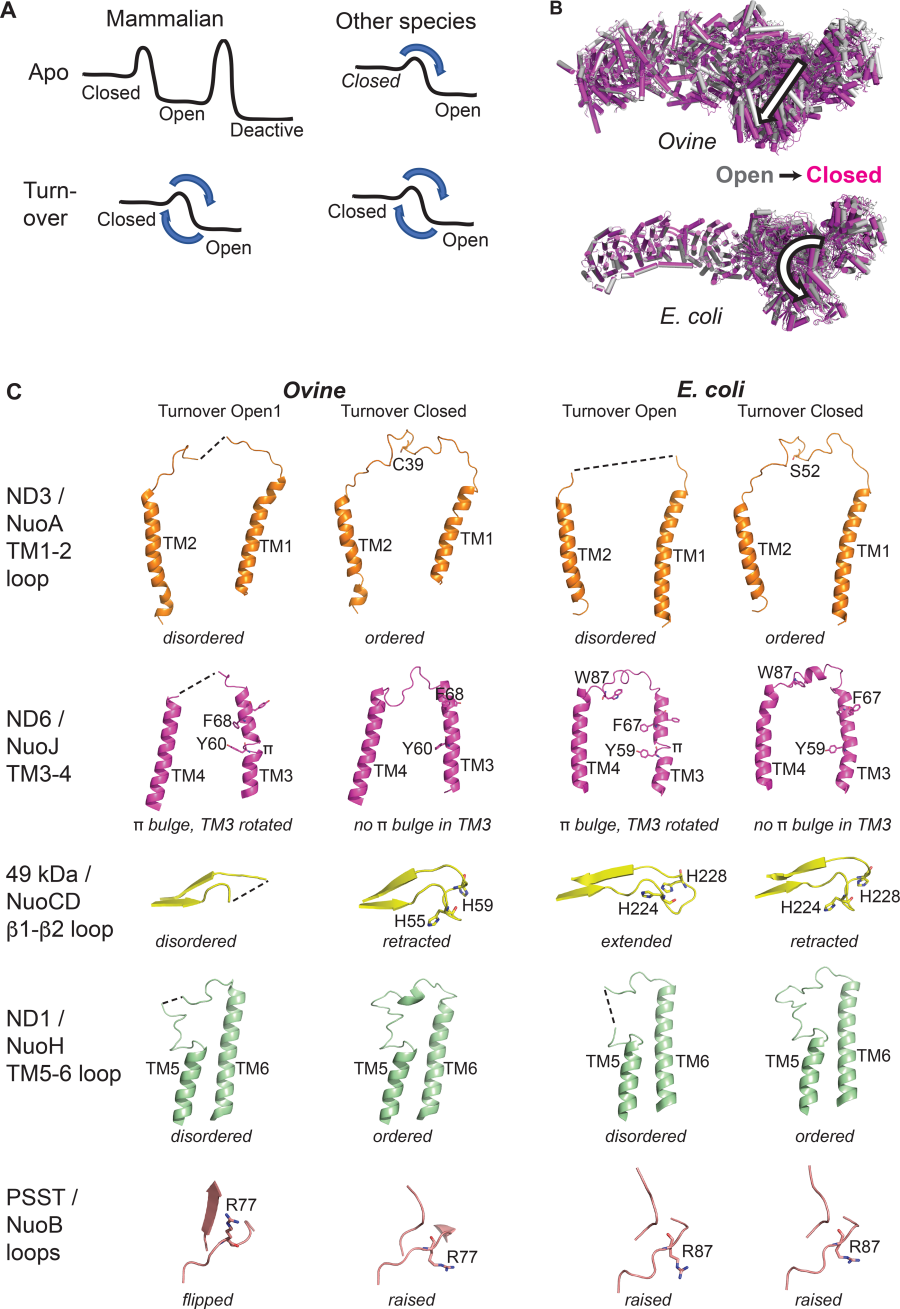
Open and closed states of complex I. (**A**) (left) The ratio of the observed closed and open states of complex I reflects relative energy levels for each state. Mammalian enzyme has large barriers between the states in apo and shows an additional, distinct, deactive state. Under turnover, due to redox reactions, the barrier between the closed/open states is lowered but both states can be observed. (right) Most other species do not show a deactive state, thus have a lower barrier between the states in apo, and so tend to ‘slip' into lower energy state. However, under turnover both states must be observed, as they are intermediates in the catalytic cycle. (**B**) View from the tip of PA of the open (grey) to closed (magenta) state transition in complex I. In the mammalian enzyme it is mostly a sideways tilt of PA in the direction of an arrow, while in *E. coli* it is an anticlockwise PA twist. (**C**) Comparison of conformations of the key loops in the open and closed states of mammalian (*Ovine*) and bacterial (*E. coli*) complex I. Ovine open states were separated into several similar sub-states [25], differing by the PA–MA angle, and open1 state is used here. Structures of parts of subunits (listed on the left) of both enzymes under turnover are shown in a similar orientation (Ovine PDB IDs 6ZKD and 6ZKC, *E. coli* PDB IDs 7Z7T and 7Z7S). Key residues discussed in the text are shown as sticks and indicated.

Already the first mammalian complex I structures revealed that it can exist in two major structural states [15,16], which we called ‘closed' and ‘open'. Initially we referred to them as such [15] due to apparently larger angle between the two arms in the open state, although it rather reflects a sideways tilting (with some twisting) movement of the PA (Figure 2B). One of the main differences between the states is a tight, fully enclosed (except for narrow exit into the lipid bilayer) quinone (Q) cavity in the closed state and a wider, open also to the matrix, cavity in the open state. A similar open-to-closed state transition in the *E. coli* enzyme (and also in *Chaetomium* [38] and *Drosophila*[39]) reflects instead of tilting mostly a twisting motion of PA (open-to-closed anticlockwise when observed from the PA tip, Figure 2B). Therefore, instead of referring to the PA–MA angle, the closed/open state terminology is still applicable to all species by referring to the state of Q cavity.

Q binding site is a long (∼25 Å) channel at the PA/MA interface, with a very narrow entry point from the lipid bilayer at one end and a headgroup-binding site at another, deep end, near cluster N2 [25]. The cavity is lined by flexible loops: 49 kDa/NuoCD β1–β2 loop, ND1/NuoH TM5–6 loop and two PSST/NuoB loops (Figure 2C). ND3/NuoA TM1–2 loop seals the cavity from the matrix and embraces PA/MA interface. Most of these loops are disordered in the open state, leading to a wider channel, which facilitates quinone movement into the cavity at the start of the catalytic cycle and quinol movement out at the end. Opening to the matrix facilitates these movements further by allowing waters to come in and out as necessary [26]. On the other hand, in the closed state all these loops re-order, so that the Q channel becomes narrow, tightly engulfing bound quinone. Matrix opening gets closed, so that two substrate protons needed for protonation of reduced Q can come only from protein (nearby 49 kDa/NuoCD His/Asp pair) and not from bulk water.

Further changes happen in the E-channel. Highly conserved ND6 TM3 in the open state has a π-bulge in the middle of helix, where key Y60 (ovine residue numbering in this section as some features are specific to mammals) interacts with other polar residues of the central axis. In this conformation, conserved large hydrophobic TM3 residues block the path for proton transfer along the axis, which is otherwise uninterrupted all the way from Q cavity to the tip of ND5 (Figure 1C). Remarkably, in the closed state half of TM3 rotates almost 180°, so that helix straightens, π-bulge disappears and its hydrophobic residues move out of the path (this feature was thus recently referred to as a π-gate [38]). This fills in the gap with waters so that continuous proton pathway is established along the entire central axis (Figure 1C). This is complemented by a dramatic flip of the conserved _ND1_Y142 (prefix indicates subunit) from its outward-facing conformation (open state) into the central proton path (closed state). This switch of tyrosine is conserved in all known open/closed structures from different species and is an easily recognisable indicator of the enzyme state.

Overall, these open-to-closed changes are highly conserved between the species, e.g. ovine and *E. coli* (Figure 2C) with only slight variations. For example, 49 kDa β1–β2 loop in closed states is always retracted from the cavity to allow Q to bind in the deep site (Q_d_) near N2. However, in the open state, this loop can be either extended into the cavity (*E. coli* turnover*,* ovine NADH reduced) or can be disordered (ovine turnover). For open-like states in other species, in *Yarrowia* [37] it appears to be retracted, but is extended in NDH complex from cyanobacteria [40,41]. Therefore, in the closed state β1–β2 loop appears to be always retracted, while in the open state it is more variable, with tendency to be either extended [26] or disordered (but likely still occupying the deep end of the cavity as Q_d_ is not observed [25]) under turnover conditions. The ND1 TM5–6 loop is always ordered in a very similar conformation in all known closed state structures, while its degree of disorder in the open state can vary (Figure 2C). The first PSST loop (residues 48–51) becomes a β-strand in the open mammalian enzyme [25] (and also in *Chaetomium*[38]) but remains a loop in bacteria [26] (Figure 2C). The second PSST loop (residues 75–80) flips in the open mammalian enzyme, so that conserved R77 completely changes orientation, which does not happen in bacteria. The rotation of _ND6_TM3 is accompanied by large movements of TM3–4 loop, which differ in detail between bacteria and mammals (Figure 2C) but the loop still communicates with key ND3 TM1–2 loop in all cases.

There are two absolutely conserved features in _ND6_TM3 and ND3 loop, which clearly define either open or closed state in all studied species. Closed state can be defined as the one without π-bulge in _ND6_TM3 (thus a connected central axis) and with ordered ND3 TM1–2 loop (thus enclosed Q cavity). Open state can be defined as the one with π-bulge in _ND6_TM3 (thus disconnected central axis) and with disordered central part of ND3 TM1–2 loop (thus opened to matrix Q cavity). Both of these features are central to the coupling mechanism as discussed below. Additionally, in the closed state all the key loops are ordered forming tight Q channel, while in the open state cavity-lining loops may be disordered to a different degree in different species, leading to enlarged on average Q cavity. It should be noted that key loops as built in the deposited PDB models for a range of species are not always well defined by cryo-EM density, so one has to consult the deposited maps to make conclusions on the conformation of these loops.

**Table 2.** Polar interactions changing conformation between closed and open states of complex I

Structural Elements	*E. coli* closed	*E. coli* open	Ovine closed	Ovine open
ND6/NuoJ TM3-4 loop	**K_R25 – J_E84**	**E moves out**	**ND4L_R23 – ND6_E81**	**E disordered**
Interactions with ND3/NuoA TM1-2 loop	A_R44 – CD_D191	R disordered, D moves away	R not conserved, but next is ND3_E32 – B14_R99	E moves away
	A_K46 – H_E71	K disordered	ND3_K33 – ND1_E59	ND3_K33 – ND1_E59
	**A_E51 – H_K140**	**E disordered**	**ND3_E38 – ND1_K126**	**E disordered**
	**A_S52 – H_Y141**	**S disordered**	**ND3_C39 – ND1_Y127**	**C disordered**
	**A_D55 – B_R112**	**D disordered**	**ND3_D42 – PSST_K102**	**D moves away**
	A_R63 – CD_D214	D moves away	R not conserved	
	A_K67 – CD_D238	K and D move apart	D not conserved	
ND1/NuoH TM5-6 loop	H_E216 sidechain is far from B_R87	E disordered	*ND1_E202 – PSST_R77*	*ND1_E202 –ND1_R279*
	**H_E220 – H_R148**	**E disordered**	**ND1_E206 – ND1_R134**	**E disordered**
	H_E220 – CD_R407	E disordered	ND1_E206 – ND1_R209	E disordered
	**H_E228 – B_R91**	**E and R move apart**	**ND1_E214 – PSST_R81**	**E and R move apart**
49kDa/NuoCD β1-2 loop	**CD_H224 – H_Y141**	**H and Y move apart**	**49kDa_H55 – ND1_Y127**	**H disordered, Y moves away**
	**CD_H228 – CD_D329**	**H moves away**	**49kDa_H59 – 49kDa_D160**	**H disordered, 49kDa_D160 - PSST_R77**
PSST/NuoB loop/strand	B_R87 - H_D223 R87 interacts with Q_mid headgroup	D moves away (not conserved) R87 does not change orientation	*PSST_R77 – ND1_E202 R77 interacts with Qs (shallow site) headgroup*	*PSST_R77 - 49kDa_D160 Loop becomes b-strand, R77 flips away from Qs to D160*

The ordering of key loops is ensured by many conserved salt bridges and polar interactions formed in the closed state, which are all broken in the open state (Table 2). Most of these interactions are conserved from bacteria to mammals, consistent with their key mechanistic role. One interesting difference is that in mammals the PSST loop flips, bringing conserved R77 to interact either with shallow site (Qs) -bound quinone in the closed state or with key _49 kDa_D160 (from the Q-protonating pair) in the open state [25]. In bacteria the homologous R87 does not flip, but it also interacts with similarly positioned Q in the closed state, suggesting conserved interactions in this intermediate Q site. Native quinone with its long tail can bind there only temporarily on the way to the deep site, while short-tail quinones used in cryo-EM experiments can occupy both sites simultaneously [25,26].

Until recently mammalian complex I was unique by showing closed and open states in apo conditions (meaning here as purified, no substrates added) [15,42]. It is also unique by showing a transition into the so-called deactive state with high-energy barrier, requiring prolonged incubation at elevated temperature (30–37°C) without substrates [43]. Deactive state resembles open state as all the key loops are disordered and there is a π-bulge in _ND6_TM3 [42]. Due to this similarity it was suggested that deactive and open state are one and the same [44]. However, ‘as purified' ovine enzyme shows mostly open state in cryo-EM analyses and yet it does not show any time delay in developing activity after addition of substrates [25]. This delay is a defining characteristic of the deactive state, as enzyme needs several cycles of slow turnover to recover from deactive conformation into active state. Thus, fully active mammalian enzyme can have various proportions of open/closed states (from mostly open to mostly closed) depending on species and purification procedure [45].

We have shown that true deactive state differs from open state by the complete relocation of ND6 TM4 [25]. It should be noted that our deposited maps for deactive states (differing by PA–MA angle) were post-processed with optimal B-factor for the bulk of molecule. Less well-ordered peripheral parts are better visualised by applying blurring B-factor of about 100 (e.g. in Coot) or by filtering density to ∼4 Å. Then the relocated ND6 TM4 density becomes very clear (e.g. PDB IDs 6ZKU, 6ZKV). The C-terminus of the lateral ND5 helix and subunit B14.7, in the vicinity of ND6 TM4, are mostly disordered in the deactive enzyme. This is however also the case for some of the more open classes of the active enzyme, including apo conditions. Together with the fact that deactive enzyme can be re-activated this suggests rather local disorder in this area in the most open (i.e. with the largest PA-MA angle) classes and not a loss of subunits/degradation. However, the relocation of ND6 TM4 is unique to deactive enzyme and is not observed in any other open classes. It puts ND6 TM3–4 loop in between PA and MA like a spanner in the works, explaining enzyme deactivation and a high barrier for active/deactive transition.

Another still often-used characteristic of the deactive state is the sensitivity of enzyme to NEM exposure, which modifies _ND3_C39 (Figure 2C). However, this cysteine is exposed (thus accessible to NEM) both in open and in deactive states (while it is tucked away into Q cavity in closed state), so NEM assay is not specific for deactive enzyme but instead indicates the proportion of open plus deactive (if any) states. The only specific biochemical assay for deactive state is the delay in developing activity, as noted above.

Structures of (apo) complex I from other species have shown so far mostly open-like states (judging by _ND6_TM3 and density for key loops) — e.g. *T. thermophilus* (PDB 6Y11), *Arabidopsis* (PDB 7AR8, 7ARB), *Polytomella* (PDB 7ARD) and NDH complex (PDB 6NBY, 6HUM). This alone would argue against considering open state as equivalent to deactive, since these species do not show any active/deactive transition. To understand this distribution of conformations, we can consider that closed–open–deactive states of mammalian enzyme are part of the same conformational trajectory with large energy barriers in between (due to a high barrier for deactive transition) (Figure 2A). Other species usually would have lower closed-to-open state barrier (as there is no deactive state) and so would ‘slip' into lower energy open state in the absence of turnover (i.e. for ‘as purified’ apo enzyme). Therefore mammalian apo enzyme can be seen in both closed and open states, while for most other species only open state can be observed. That is however, not always the case, as *Chaetomium* [38] and *Drosophila* [39] apo enzymes (both lacking deactive transition) were recently observed in both states, probably due to higher energy barrier between closed and open states in these species. These observations demonstrated that open/closed states are not unique for mammalian enzyme. For species showing only open apo state it would be expected that under turnover a closed state would appear, since if open/closed transition is a part of catalytic cycle then closed state should be populated to some degree during turnover (Figure 2A). This is exactly what we observed with *E. coli* enzyme [26], finally settling the debate. It is now clear that open/closed transitions are part of catalytic cycle in all species, while the deactive state is an ‘extreme' stabilised form of open state, specific for mammalian enzyme, where it may be important in preventing ischemia/reperfusion injury [46].

One distinct example requiring further clarification is *Yarrowia* enzyme. It is the only other species, apart from *E. coli* and ovine, studied under turnover so far. However, both apo and turnover states were observed to be open-like, judging by _ND6_TM3 and ND3 loop state [37]. *Yarrowia* enzyme ‘as purified’ (apo) exists in a ‘low barrier' deactive state, from which it can be quickly reactivated. It is distinct from mammalian deactive state as key Q loops are mostly ordered and there is no relocation of _ND6_TM4 [37]. Under turnover ND1 loop reforms from apo conformation, accommodating Q binding, but ND3 loop is disordered and π-bulge in _ND6_TM3 remains, indicating that the single dominant observed under turnover *Yarrowia* state is open. The ratio of closed-to-open states under turnover can be variable, from 10 [25] to 54% [26] closed for ovine or 4 to 24% closed for *E. coli* [26]. This reflects relative energy levels of each state under turnover, depending on conditions (pH) and species. Since minor 3D classes (such as 5–10% closed) are challenging to classify out with standard cryo-EM techniques, it is likely that further processing, perhaps with our focus-reverse-classify approach, developed specifically for this purpose [47], will reveal closed state in *Yarrowia* as well.

## Mechanism of coupling between electron transfer and proton translocation

Since we established that complex I cycles between open and closed conformations during turnover, and we had high-resolution structures (with waters resolved) of these states from two evolutionary distant species (ovine [25] and *E. coli* [26]), the stage for developing a first experiment-based complex I mechanism was set. The analysis of structures brought many surprises, contradicting previous assumptions. First, despite large conformational changes around Q cavity and E-channel, discussed above, there was virtually no difference in the conformations of ALS subunits between open and closed states, including, crucially, during turnover, in both species. This rules out previous mechanisms where conformation/solvent exposure of proton channels in ALS was proposed to change. Instead, data strongly supports a purely electrostatic mechanism for the ALS, as also indicated by apparent changes in the protonation state of some key residues. Several glutamates in the E-channel consistently become charged (side chain cryo-EM density disappears) in closed state [25,26].

Another surprise from the recent structures is the unique hydration pattern of the ALS, with the ND5 being much more hydrated at the IMS/periplasm side than ND2/ND4, first observed in mammalian enzyme [25] and later in yeast [37] and *E. coli* [26]. This unique hydration profile is consistent with all available complex I and NDH structures, as well as with molecular dynamics simulations [37], although in one case conclusions differed [48]. This suggests that ND2 and ND4, lacking any polar residues or waters at the IMS side, do not possess viable hydrated proton pathways leading towards IMS and only ND5 has a full proton input and output pathway (Figure 1C). _ND5_HisTM8 may rotate on the TM8 π-bulge to establish a connection either towards the rest of the central axis or towards _ND5_GluTM12, thus re-distributing incoming protons and preventing proton back-leak. ND4 appears also to have proton input pathway from matrix/cytosol with a similar switch/gate at _ND4_LysTM8 [26]. ND2 probably does not have such an input (Figure 1C), but this may depend on species, and whether ND2 has matrix input pathway or not is not important for our mechanism, which will work in both cases. The E-channel is dry on both sides of the membrane, ruling out its direct involvement in proton pumping. This suggested an unexpected possibility that all the protons are ejected through ND5. This model is consistent with a very distinct sequence conservation pattern of ND5, conserved all along input, central axis connection and output pathways, while ND2/ND4 are conserved mostly along the central axis.

On the basis of this new structural knowledge we have developed a novel coupling mechanism of complex I, first proposed for mammalian enzyme [25], and then further elaborated and refined using *E. coli* data [26]. We set out to explain, on the basis of minimal assumptions, all the unusual structural features of complex I, including open-to-closed transition and ND5-only proton exit. After some permutations, we eventually arrived at a very robust and straightforward, in our opinion, ‘domino effect' mechanism, which naturally accounts for (and is based on) these unexpected new findings.

**Figure 3.**
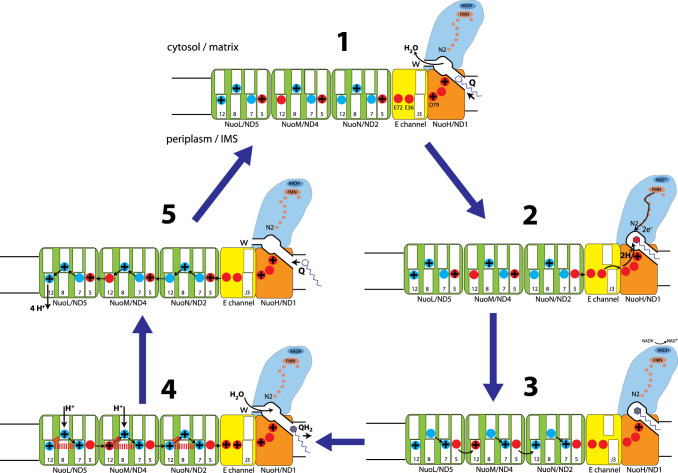
A ‘domino effect' coupling mechanism of complex I. An overview of the proposed complex I mechanism. Individual steps involve conformational changes around Q cavity/E-channel and electrostatic interactions in antiporters NuoL/M/N, as described in the text. Complex I cycles between the open state (Steps 1, 4 and 5), where the Q cavity is widened and opened both to the lipid bilayer (Q) and to the cytosol (W), and the closed state (Steps 2 and 3), where the Q cavity is enclosed and tightly engulfs the bound quinone, sealed by the quinone tail. NADH oxidation and electron transfer in PA in Step 2 are fast and not rate-limiting. Charged quinone intermediate is indicated by the red headgroup, and quinol by the grey-filled headgroup. The charged residues on the MA central axis are indicated in blue for lysines and in red for glutamates/aspartates. For clarity, the protonated forms are shown with a + sign and un-protonated are empty (although the actual charge would be +/0 for lysine and 0/− for glutamate/aspartate). The key helices in antiporters are indicated by their numbers. In the open state the water wire between the Q cavity and the central axis in the E-channel is broken at _J_TM3 (indicated as J3). The connection is established in the closed state due to _J_TM3 rotation. Black arrows indicate proton transfer, including re-distribution along the central axis. Access from the cytosol happens via NuoL/M and the exit into the periplasm only via NuoL. Electrostatic interactions, resulting in the ejection of four protons into periplasm in Step 5, are indicated as red dashes in Step 4. Reproduced from [26] with permission.

First, we consider that in open state Q cavity widens and opens to matrix (site W in Figure 3), although the entry site from lipids (site Q in Figure 3) remains narrow. Quinone is observed bound in shallow/median sites near the entrance but not at the deep site near N2, prevented by the extended/disordered 49 kDa/NuoCD β1–β2 loop. Therefore we propose that in open state quinone comes in or quinol comes out of cavity, and opening to the matrix is necessary to allow accompanying waters to come out/in to fill the cavity, as quinone tail would block the narrow lipid entry point. In closed state the cavity is fully enclosed and tightly engulfs quinone, which is now bound in the deep site. In this state quinone cannot move but can accept electrons from N2. The two protons needed to finish the reaction come from nearby 49 kDa His59-Asp160 (NuoCD His228–Asp329) pair, as only a few remaining in cavity waters cannot provide them. The cavity is enclosed on all sides except a connection through a funnel of charged residues to the E-channel, and then only in closed state it is connected further to the rest of central MA axis. This provides a natural way for redox reaction to initiate proton translocation events, because the two protons needed to re-protonate 49 kDa/NuoCD His/Asp pair have to come from the central axis (as evidenced by de-protonation of several E-channel glutamates).

Second, the ND5-only proton exit, along with the absence of any conformational changes in ALS, lead us to realise that this actually ensures tight ALS communication to each other in a series of proton transfer events, which must happen, unexpectedly, mostly along the central MA axis.

The proposed [26] cycle (Figure 3 and Supplementary Movie S1) starts with **Step 1**, where quinone temporarily binds in shallow/median site. Site W is open for waters to flow out of the cavity and give space to the incoming quinone. In this state the ALS are maximally protonated at the key *TM8* sites (Lys/HisTM8) by protons coming into NuoL/NuoM (for simplicity in the description of the cycle we use only *E. coli* nomenclature/residue numbering as shown in Figure 1C) from the cytoplasm and redistributed along the central axis until the _NuoJ_TM3 break. For the ease of following proton transfers we indicate any protonated residues just by a ‘+' sign, leaving unprotonated ones empty. The break prevents futile proton leak into the Q cavity and back to the cytosol. For the closely interacting *TM7/TM5* ion pair sites (LysTM7/GluTM5), the proton is proposed to reside in *TM5* site, as suggested by cryo-EM observations [25,26]. The residues in *TM7/TM5* ion pairs are thus uncharged and interact weakly. The *TM12* (Lys/GluTM12) sites have a lower *pK*a and tuned to remain unprotonated in this state due to electrostatic interactions with protonated *TM8* and *TM5* sites. The exception is NuoL *TM12* site, which is protonated as it is distal and so does not have a *TM5* partner. **(Step 2)** Bound quinone traverses into the deep site, triggering the open-to-closed transition, so that the W site is closed off and the Q cavity tightly engulfs quinone. _NuoJ_TM3 rotates, establishing the uninterrupted proton path from the Q cavity all the way to the MA tip. Quinone accepts two electrons from cluster N2, and the unstable charged quinone intermediate is immediately protonated by the coordinating His/Asp pair. Since the Q cavity is sealed, the protons for the re-protonation of His/Asp pair come from the central axis (_H_D79 and _N_GluTM5). **(Step 3)** In a ‘minimal' interpretation (Occam's razor) of the subsequent events, de-protonation of these residues first triggers proton transfer from *TM8* to *TM7* site in NuoN, due to the removal of large positive charge around *TM5* area. In a series of ‘domino effect' events, the removal of _N_*TM8* charge allows _M_*TM5* proton to hop on _N_*TM12* site, repeated in NuoM/L by _M_*TM8* to _M_*TM7*, _L_*TM5* to _M_*TM12* and _L_*TM8* to _L_*TM7* hops (indicated by black arrows in Figure 3 Step 3). The exact sequence of each proton hop is given in Supplementary Movie S1. De-wetting of the *TM8* area due to de-protonation, as observed in MD simulations [49], would prevent the back-flow of protons (along with possible role of _L_HisTM8/_M_LysTM8 switches). Effectively, due to the ‘forcefully’ protonated *TM12* sites and a shift of proton from *TM5* to *TM7* sites (so that the residues in *TM7/TM5* ion pairs are now charged and interact strongly) the enzyme will now be in a highly energised state, akin to stacked dominos ready to fall. **(Step 4)** The presence of the freshly produced quinol in the deep site along with the re-protonated state of coordinating residues triggers the transition from the closed to the open state, so that the Q cavity widens and the W site opens, allowing waters to come in and help quinol on its way out. The *TM8* sites (and _K_E72/E36) can be fully protonated from the cytosol, blocked off from the Q cavity by _J_TM3. In total at this stage six protons (four to be pumped and two substrate) will enter the central axis. Five of them can enter via NuoL/M, re-distributed along the central axis to *TM8* sites in NuoL, M and N, as well as to _K_E72 and _K_E36, while the sixth proton for _H_D79 can come via open Q cavity. In this scenario the re-protonation of these sites is rather ‘passive', the key to coupling being that *TM12* and *TM7/TM5* sites state is fully controlled by quinone reactions. **(Step 5)** Electrostatic interactions with the protonated *TM8* and *TM7* sites (red dashes in Step 4) lead to a large decrease in *pK*a's of *TM12* residues, forcing them to lose their protons. In NuoL the *TM12* proton would be ejected directly into the periplasm/IMS. In the reverse wave of the ‘domino effect' this will initiate a sequence of proton hops from _L_*TM8* to _L_*TM12*, _L_*TM7* to _L_*TM8* and _M_*TM12* to _L_*TM5*, repeating twice more in NuoM/N and ending with _K_E72 donating proton to _N_*TM5*. The simple natural basis for this transfer of protons along the central axis is the appearance of a ‘vacancy' on the ‘left' of the chain and the electrostatic ‘pressure' of the incoming proton from the ‘right' (or reverse in Step 3). Effectively, after the cycle is repeated three times, each time ending closer to NuoL, in the end the _M_*TM12*, _N_*TM12*, _K_E72 and _K_E36 sites would transfer their protons along the central axis towards NuoL, adding up as the four protons ejected into the periplasm (Supplementary Movie S1). This brings the system back to Step 1, with *TM8* protonated and a proton in *TM7/TM5* ‘switch' sitting again on *TM5*, thus the cycle re-starts. Crucially, for the mechanism to work, a *TM12* proton from NuoN/M must be transferred to the neighbouring NuoM/L *TM5* and not directly to the periplasm, as otherwise the process will not be initiated in the next subunit (i.e. a domino will fall without tripping the next one). Similarly, in Step 3 it is essential for protons to hop across subunit interfaces from *TM5* to *TM12* sites. Therefore, our mechanism naturally explains initially counter-intuitive NuoL-only exit. The pump works with protons moving along the entire central axis either towards Q cavity (Steps 2–3) or in the reverse wave (Step 5), thus the periplasm/IMS side must be shielded from the solvent everywhere except the NuoL exit.

The mechanism also explains the reverse electron transport in complex I: under these conditions high pmf and a reduced quinone/quinol pool would promote reverse reaction by driving charge transitions in ALS in reverse to those in Figure 3. Translocation of protons into the matrix (the reversal of Steps 5 and 4, driven by high pmf and accompanied by quinol entering the cavity) would be coupled to the transfer of protons from the Q coordinating residues into the central axis (the reversal of Steps 3 and 2), creating a double negative charge near Q deep site. It would promote quinol binding as well as lower the N2 redox potential, enabling reverse electron transfer from N2 to FMN and NAD^+^. The presence of ready proton acceptors in the form of de-protonated Q coordinating residues will allow the quinol oxidation reaction to complete.

The ‘domino effect' mechanism is straightforward, robust and naturally explains the NuoL-only proton exit, why the Q entry site is so narrow, why the W site exists and why ND3/NuoJ TM3 rotates. The arrangement of key *TM12*, *TM8* and *TM7/TM5* sites appears to be a minimum necessary to allow for the mechanism.

Despite NuoL-only exit, all three ALS and the E-channel are essential, being responsible for the eventual transfer of one pumped proton each. Therefore, the varying number of ALS is related to the number of protons pumped per cycle in each of evolutionary-related complexes, such as MRP [34] and membrane-bound hydrogenases, according to the available redox energy [50]. The mechanism appears to be conserved in these proteins: the Q-like cavity encloses different substrates, such as sodium ions, plastoquinone, hydrogen or polysulfide, but the principle of the redox charge action via the lateral proton transfer along the central axis remains fully applicable [34].

## Remaining questions and future work

Recent cryo-EM structures provided a strong experimental basis for our detailed complex I mechanism (Figure 3), which was published only a few months ago [26] and so it remains to be seen whether alternative proposals will continue to proliferate as intensively as they were until now or whether a wider consensus will start to emerge in the field.

Our initial proposal on ND5-only proton exit [25] was supported by experimental work and MD simulations on *Yarrowia* enzyme [37], as well as on a related MRP antiporter [51]. On the other hand, others did not agree [48], even though MD data seem to indicate very little, if any, hydration on the IMS side in ND4, ND2 and E-channel, in stark contrast with ND5 (Figure 2A in [48]).

The details of the coupling mechanism itself are also very different in recent proposals. In *Yarrowia* work [37] the mechanism involves charged quinone intermediates, which shuttle twice per cycle between deep and shallow sites. However, such long-lived intermediates are energetically unfavourable and have not been observed experimentally [52]. The interactions between chemical and pumped protons at the putative proton-loading site (PLS) near the Q shallow site would require fine tuning and ‘orchestration' of events to ‘push' protons into E-channel. Also, it is not clear how those two ‘pushed' protons would drag along another two pumped protons. Overall, the mechanism seems to be unnecessary complicated and potentially ineffective (moving quinone twice per cycle instead of once would lose energy). In another proposal, stemming from MD simulations, a single proton is also ‘pushed' from the shallow Q site into E-channel but it drives the entire cycle [49,53]. Here, in the ‘forward' wave, ion pairs in ALS ‘open', shifting protons from key TM8 to TM12 residues. The ‘reverse' wave is then imitated by proton release from ND5/NuoL to IMS, so that TM8 residues are re-protonated from the matrix, ion pairs close and each ALS (and E-channel) pumps out one proton each. Here, again, using just one proton to initiate the whole cycle seems ineffective, putting a lot of strain on a single event. There are some similarities with our mechanism as the forward wave energises the MA and the reverse wave releases protons into IMS, however, the direction and sequence of proton transfer events are completely different. And of course the mechanism is not consistent with growing consensus on ND5-only output.

It should be noted that a putative mechanism for proton translocation in complex I, also termed ‘domino effect', was suggested earlier [54]. However, despite some similarity involving consecutive electrostatic interactions between the ALS, the basic principles there were very different from our mechanism, as proton input/output was proposed to happen individually across each ALS for each pumped proton and proton transfers between ALS and a single NuoL output, a key to our mechanism, were not envisaged.

The debate on whether the open state and deactive state of mammalian complex I are one and the same is now settled, in our opinion, with, among others, recent publications on *E. coli* [26], *Chaetominum* [38] and *Drosophila* [39]. It is clear that both closed and open states are part of the catalytic cycle by active enzyme, while deactive (mammalian) and resting (bacterial, with disrupted Q cavity) [26] states are special off-pathway states. In a recent review, in an attempt to explain experimental observations, it was suggested that different species may enter a deactive-like state either rarely (WT mouse) or often (ND6 P25L mouse mutant) during catalysis, depending on how fast relaxing they are into deactive state [44]. However, it was still concluded that only closed complex I is active in the catalytic cycle and any open/deactive states are off-pathway. This is hard to imagine mechanistically, as a long quinone molecule would have to squeeze in and out of very narrow Q passage ∼200 times per second. The ‘only closed complex is active' notion also contradicts a growing number of examples of species showing closed/open states but no active/deactive transition. It also contradicts the striking fact that *E. coli* (with more examples from other species likely to follow) shows closed state only during turnover, otherwise being in a mixture of open/resting states [26].

What experiments can be done to clarify any remaining disputes/questions? Clearly, more cryo-EM data from enzyme undergoing turnover needs to be collected from more species. Only such experiments can reveal a full repertoire of 3D conformations of the complex. It is important to process the data carefully and extensively, as minor 3D states can be difficult to identify in the routine classification. Here, either our focus-reverse-classify approach [47] or the latest 3D classification methods in Cryosparc, focused on the E-channel/Q cavity area [39], will be useful. Most of species adopt open state in apo, but some show mostly closed state, e.g. *Tetrahymena* [55], mouse [42], and possibly *Paracoccus denitrificans* [44]. For these species it is likely that open state will appear under turnover, which would be an important result. It is possible that pH of the buffer, in which turnover is performed before flash-freeze for EM grids, may need to be adapted. We have observed that, consistent with de-protonation of key E-channel residues in the closed state, the proportion of this state is increased with pH [26]. Therefore, if the proportion of the minor open state needs to be increased (either in apo or during turnover), the pH should be decreased to ∼pH 5, and vice versa (pH 8–9 can be used to promote closed state).

The results of extensive mutagenesis experiments performed with *E. coli* and other species [26] are strongly consistent with our mechanism, but do not fully differentiate it from others, as e.g. central MA axis residues are essential in any mechanism. Perhaps introducing periplasm-side proton exits into ND4/ND2 by mutations into polar residues by homology with ND5 may help – it should not interfere with ‘one-proton-per-ALS' mechanism, but would disrupt our mechanism. The importance of sealing the Q cavity in the closed state may be verified by mutations of the central part of ND3 loop with an aim to introduce leaks, which should abolish proton pumping. Some residues around NuoJ/ND6 TM3 p-bulge were mutated, with loss of activity and proton pumping in *E. coli* V65G mutant [56]. Another important residue to mutate here is V63, which blocks the proton path in the open state. Its mutations to Gly or Ala may re-connect the path in the open state, leading to proton leaks into the Q cavity and disrupting proton pumping. One of the key residues not yet mutated is NuoL H254 from TM8, which links proton input path with the central axis (Figure 1C).

More direct ways to observe any movements in complex I (such as open/closed transitions during turnover) are experimentally challenging but are becoming feasible. One possibility is to use high-speed atomic force microscopy (AFM) to image movies of single complex I molecules, absorbed on mica sheets, during turnover. The methodology currently approaches required special resolution (∼5 Å) to resolve potential movements of the PA tip, although temporal resolution (∼100 ms) [57] will need to be improved to match complex I turnover rate (∼5 ms). Alternatively, the sample may be imaged cooled down (as we did for mammalian enzyme [25]) or with very limiting substrate concentrations to slow down turnover. Another approach could be to label appropriate residues on the surface of PA and MA so that single-molecule FRET signals [58] can be recorded and compared in the absence and presence of turnover. Finally, time-resolved cryo-EM methods are now approaching ∼5 ms resolution [59,60] and could be applied to visualise first steps in complex I reaction after addition of substrates. However, achieving necessary temporal synchronisation of the reaction after mixing with substrates may be challenging. It could be better pursued using e.g. caged NADH and laser light to initiate the reaction, which can also potentially achieve microsecond time resolution [61].

## Abbreviations

ALS: antiporter-like subunits
FMN: flavin-mononucleotide
IMS: inter-membrane space
MA: membrane arm
PA: peripheral arm
TM: transmembrane

## References

[1] Nicholls, D.G. and S.J. and Ferguson, (2013) Bioenergetics 4, 4th edn. Elsevier. 419 p

[2] Moser, C.C., Farid, T.A., Chobot, S.E. and Dutton, P.L. (2006) Electron tunneling chains of mitochondria. Biochim. Biophys. Acta 1757, 1096–1109 10.1016/j.bbabio.2006.04.015 16780790

[3] Pinke, G., Zhou, L. and Sazanov, L.A. (2020) Cryo-EM structure of the entire mammalian F-type ATP synthase. Nat. Struct. Mol. Biol. 27, 1077–1085 10.1038/s41594-020-0503-8 32929284

[4] Vercellino, I. and Sazanov, L.A. (2021) The assembly, regulation and function of the mitochondrial respiratory chain. Nat. Rev. Mol. Cell Biol. 23, 141–161 10.1038/s41580-021-00415-0 34621061

[5] Iwata, S., Lee, J.W., Okada, K., Lee, J.K., Iwata, M., Rasmussen, B. et al. (1998) Complete structure of the 11-subunit bovine mitochondrial cytochrome bc_1_ complex. Science 281, 64–71 10.1126/science.281.5373.64 9651245

[6] Tsukihara, T., Aoyama, H., Yamashita, E., Tomizaki, T., Yamaguchi, H., Shinzawa-Itoh, K. et al. (1996) The whole structure of the 13-subunit oxidized cytochrome c oxidase at 2.8 A. Science 272, 1136–1144 10.1126/science.272.5265.1136 8638158

[7] Abrahams, J.P., Leslie, A.G., Lutter, R. and Walker, J.E. (1994) Structure at 2.8 A resolution of F1-ATPase from bovine heart mitochondria. Nature 370, 621–628 10.1038/370621a0 8065448

[8] Hofhaus, G., Weiss, H. and Leonard, K. (1991) Electron microscopic analysis of the peripheral and membrane parts of mitochondrial NADH dehydrogenase (complex I). J. Mol. Biol. 221, 1027–1043 10.1016/0022-2836(91)80190-6 1834851

[9] Sazanov, L.A. and Walker, J.E. (2000) Cryo-electron crystallography of two sub-complexes of bovine complex I reveals the relationship between the membrane and peripheral arms. J. Mol. Biol. 302, 455–464 10.1006/jmbi.2000.4079 10970745

[10] Baranova, E.A., Holt, P.J. and Sazanov, L.A. (2007) Projection structure of the membrane domain of *Escherichia coli* respiratory complex I at 8 Ă resolution. J. Mol. Biol. 366, 140–154 10.1016/j.jmb.2006.11.026 17157874

[11] Sazanov, L.A., Carroll, J., Holt, P., Toime, L. and Fearnley, I.M. (2003) A role for native lipids in the stabilization and two-dimensional crystallization of the *Escherichia coli* NADH-ubiquinone oxidoreductase (complex I). J. Biol. Chem. 278, 19483–19491 10.1074/jbc.M208959200 12637579

[12] Sazanov, L.A. and Hinchliffe, P. (2006) Structure of the hydrophilic domain of respiratory complex I from *Thermus thermophilus*. Science 311, 1430–1436 10.1126/science.1123809 16469879

[13] Efremov, R.G. and Sazanov, L.A. (2011) Structure of the membrane domain of respiratory complex I. Nature 476, 414–420 10.1038/nature10330 21822288

[14] Baradaran, R., Berrisford, J.M., Minhas, G.S. and Sazanov, L.A. (2013) Crystal structure of the entire respiratory complex I. Nature 494, 443–448 10.1038/nature11871 23417064 PMC3672946

[15] Fiedorczuk, K., Letts, J.A., Degliesposti, G., Kaszuba, K., Skehel, M. and Sazanov, L.A. (2016) Atomic structure of the entire mammalian mitochondrial complex I. Nature 538, 406–410 10.1038/nature19794 27595392 PMC5164932

[16] Zhu, J., Vinothkumar, K.R. and Hirst, J. (2016) Structure of mammalian respiratory complex I. Nature 536, 354–358 10.1038/nature19095 27509854 PMC5027920

[17] Gutierrez-Fernandez, J., Kaszuba, K., Minhas, G.S., Baradaran, R., Tambalo, M., Gallagher, D.T. et al. (2020) Key role of quinone in the mechanism of respiratory complex I. Nat. Commun. 11, 4135 10.1038/s41467-020-17957-0 32811817 PMC7434922

[18] Verkhovskaya, M. and Bloch, D.A. (2013) Energy-converting respiratory complex I: on the way to the molecular mechanism of the proton pump. Int. J. Biochem. Cell Biol. 45, 491–511 10.1016/j.biocel.2012.08.024 22982742

[19] Wikstrom, M., Sharma, V., Kaila, V.R., Hosler, J.P. and Hummer, G. (2015) New perspectives on proton pumping in cellular respiration. Chem. Rev. 115, 2196–2221 10.1021/cr500448t 25694135

[20] Kaila, V.R.I. (2018) Long-range proton-coupled electron transfer in biological energy conversion: towards mechanistic understanding of respiratory complex I. J. R. Soc. Interface 15, 20170916 10.1098/rsif.2017.0916 29643224 PMC5938582

[21] Sazanov, L.A. (2015) A giant molecular proton pump: structure and mechanism of respiratory complex I. Nat. Rev. Mol. Cell Biol. 16, 375–388 10.1038/nrm3997 25991374

[22] Zickermann, V., Wirth, C., Nasiri, H., Siegmund, K., Schwalbe, H., Hunte, C. et al. (2015) Structural biology. Mechanistic insight from the crystal structure of mitochondrial complex I. Science 347, 44–49 10.1126/science.1259859 25554780

[23] Agip, A.A., Blaza, J.N., Fedor, J.G. and Hirst, J. (2019) Mammalian respiratory complex I through the lens of cryo-EM. Annu. Rev. Biophys. 48, 165–184 10.1146/annurev-biophys-052118-115704 30786232

[24] Haapanen, O. and Sharma, V. (2018) A modeling and simulation perspective on the mechanism and function of respiratory complex I. Biochim. Biophys. Acta Bioenerg. 1859, 510–523 10.1016/j.bbabio.2018.04.001 29660310

[25] Kampjut, D. and Sazanov, L.A. (2020) The coupling mechanism of mammalian respiratory complex I. Science 370, eabc4209 10.1126/science.abc4209 32972993

[26] Kravchuk, V., Petrova, O., Kampjut, D., Wojciechowska-Bason, A., Breese, Z. and Sazanov, L. (2022) A universal coupling mechanism of respiratory complex I. Nature 609, 808–814 10.1038/s41586-022-05199-7 36104567

[27] Stroud, D.A., Surgenor, E.E., Formosa, L.E., Reljic, B., Frazier, A.E., Dibley, M.G. et al. (2016) Accessory subunits are integral for assembly and function of human mitochondrial complex I. Nature 538, 123–126 10.1038/nature19754 27626371

[28] Hinchliffe, P. and Sazanov, L.A. (2005) Organization of iron-sulfur clusters in respiratory complex I. Science 309, 771–774 10.1126/science.1113988 16051796

[29] Holt, P.J., Efremov, R.G., Nakamaru-Ogiso, E. and Sazanov, L.A. (2016) Reversible FMN dissociation from *Escherichia coli* respiratory complex I. Biochim. Biophys. Acta 1857, 1777–1785 10.1016/j.bbabio.2016.08.008 27555334

[30] de Vries, S., Dorner, K., Strampraad, M.J. and Friedrich, T. (2015) Electron tunneling rates in respiratory complex I are tuned for efficient energy conversion. Angew. Chem. Int. Ed. Engl. 54, 2844–2848 10.1002/anie.201410967 25600069 PMC4506566

[31] Pohl, T., Bauer, T., Dorner, K., Stolpe, S., Sell, P., Zocher, G. et al. (2007) Iron-sulfur cluster N7 of the NADH:ubiquinone oxidoreductase (complex I) is essential for stability but not involved in electron transfer. Biochemistry 46, 6588–6596 10.1021/bi700371c 17489563

[32] Verkhovskaya, M.L., Belevich, N., Euro, L., Wikstrom, M. and Verkhovsky, M.I. (2008) Real-time electron transfer in respiratory complex I. Proc. Natl Acad. Sci. U.S.A. 105, 3763–3767 10.1073/pnas.0711249105 18316732 PMC2268814

[33] Jones, A.J., Blaza, J.N., Varghese, F. and Hirst, J. (2017) Respiratory complex I in Bos Taurus and *Paracoccus denitrificans* pumps four protons across the membrane for every NADH oxidized. J. Biol. Chem. 292, 4987–4995 10.1074/jbc.M116.771899 28174301 PMC5377811

[34] Steiner, J. and Sazanov, L. (2020) Structure and mechanism of the Mrp complex, an ancient cation/proton antiporter. eLife 9, e59407 10.7554/eLife.59407 32735215 PMC7419157

[35] Sharma, V., Belevich, G., Gamiz-Hernandez, A.P., Rog, T., Vattulainen, I., Verkhovskaya, M.L. et al. (2015) Redox-induced activation of the proton pump in the respiratory complex I. Proc. Natl Acad. Sci. U.S.A. 112, 11571–11576 10.1073/pnas.1503761112 26330610 PMC4577180

[36] Brandt, U. (2011) A two-state stabilization-change mechanism for proton-pumping complex I. Biochim. Biophys. Acta 1807, 1364–1369 10.1016/j.bbabio.2011.04.006 21565159

[37] Parey, K., Lasham, J., Mills, D.J., Djurabekova, A., Haapanen, O., Yoga, E.G. et al. (2021) High-resolution structure and dynamics of mitochondrial complex I-Insights into the proton pumping mechanism. Sci. Adv. 7, eabj3221 10.1126/sciadv.abj3221 34767441 PMC8589321

[38] Laube, E., Meier-Credo, J., Langer, J.D. and Kühlbrandt, W. (2022) Conformational changes in mitochondrial complex I of the thermophilic *eukaryote Chaetomium thermophilum*. Sci. Adv. 8, eadc9952 10.1126/sciadv.adc9952 36427319 PMC9699679

[39] Padavannil, A., Murari, A., Rhooms, S.-K., Owusu-Ansah, E. and Letts, J.A. (2022) Resting mitochondrial complex I from *Drosophila melanogaster* adopts a helix-locked state. bioRxiv 2022.11.01.514701 10.1101/2022.11.01.514701 PMC10036122 36952377

[40] Schuller, J.M., Birrell, J.A., Tanaka, H., Konuma, T., Wulfhorst, H., Cox, N. et al. (2019) Structural adaptations of photosynthetic complex I enable ferredoxin-dependent electron transfer. Science 363, 257–260 10.1126/science.aau3613 30573545

[41] Laughlin, T.G., Bayne, A.N., Trempe, J.F., Savage, D.F. and Davies, K.M. (2019) Structure of the complex I-like molecule NDH of oxygenic photosynthesis. Nature 566, 411–414 10.1038/s41586-019-0921-0 30742075

[42] Agip, A.A., Blaza, J.N., Bridges, H.R., Viscomi, C., Rawson, S., Muench, S.P. et al. (2018) Cryo-EM structures of complex I from mouse heart mitochondria in two biochemically defined states. Nat. Struct. Mol. Biol. 25, 548–556 10.1038/s41594-018-0073-1 29915388 PMC6054875

[43] Kotlyar, A.B. and Vinogradov, A.D. (1990) Slow active/inactive transition of the mitochondrial NADH-ubiquinone reductase. Biochim. Biophys. Acta 1019, 151–158 10.1016/0005-2728(90)90137-S 2119805

[44] Chung, I., Grba, D.N., Wright, J.J. and Hirst, J. (2022) Making the leap from structure to mechanism: are the open states of mammalian complex I identified by cryoEM resting states or catalytic intermediates? Curr. Opin. Struct. Biol. 77, 102447 10.1016/j.sbi.2022.102447 36087446 PMC7614202

[45] Kampjut, D. and Sazanov, L.A. (2022) Structure of respiratory complex I - an emerging blueprint for the mechanism. Curr. Opin. Struct. Biol. 74, 102350 10.1016/j.sbi.2022.102350 35316665 PMC7613608

[46] Chouchani, E.T., Pell, V.R., Gaude, E., Aksentijevic, D., Sundier, S.Y., Robb, E.L. et al. (2014) Ischaemic accumulation of succinate controls reperfusion injury through mitochondrial ROS. Nature 515, 431–435 10.1038/nature13909 25383517 PMC4255242

[47] Letts, J.A., Fiedorczuk, K., Degliesposti, G., Skehel, M. and Sazanov, L.A. (2019) Structures of respiratory supercomplex I + III2 reveal functional and conformational crosstalk. Mol. Cell 75, 1131–1146.e6 10.1016/j.molcel.2019.07.022 31492636 PMC6926478

[48] Röpke, M., Saura, P., Riepl, D., Pöverlein, M.C. and Kaila, V.R.I. (2020) Functional water wires catalyze long-range proton pumping in the mammalian respiratory complex I. J. Am. Chem. Soc. 142, 21758–21766 10.1021/jacs.0c09209 33325238 PMC7785131

[49] Muhlbauer, M.E., Saura, P., Nuber, F., Di Luca, A., Friedrich, T. and Kaila, V.R.I. (2020) Water-gated proton transfer dynamics in respiratory complex I. J. Am. Chem. Soc. 142, 13718–13728 10.1021/jacs.0c02789 32643371 PMC7659035

[50] Yu, H., Schut, G.J., Haja, D.K., Adams, M.W.W. and Li, H. (2021) Evolution of complex I-like respiratory complexes. J. Biol. Chem. 296, 100740 10.1016/j.jbc.2021.100740 33957129 PMC8165549

[51] Lee, Y., Haapanen, O., Altmeyer, A., Kühlbrandt, W., Sharma, V. and Zickermann, V. (2022) Ion transfer mechanisms in Mrp-type antiporters from high resolution cryoEM and molecular dynamics simulations. Nat. Commun. 13, 6091 10.1038/s41467-022-33640-y 36241630 PMC9568556

[52] Wright, J.J., Fedor, J.G., Hirst, J. and Roessler, M.M. (2020) Using a chimeric respiratory chain and EPR spectroscopy to determine the origin of semiquinone species previously assigned to mitochondrial complex I. BMC Biol. 18, 54 10.1186/s12915-020-00768-6 32429970 PMC7238650

[53] Röpke, M., Riepl, D., Saura, P., Di Luca, A., Mühlbauer, M.E., Jussupow, A. et al. (2021) Deactivation blocks proton pathways in the mitochondrial complex I. Proc. Natl Acad. Sci. U.S.A. 118, e2019498118 10.1073/pnas.2019498118 34272275 PMC8307655

[54] Hummer, G. and Wikström, M. (2016) Molecular simulation and modeling of complex I. Biochim. Biophys. Acta 1857, 915–921 10.1016/j.bbabio.2016.01.005 26780586

[55] Zhou, L., Maldonado, M., Padavannil, A., Guo, F. and Letts, J.A. (2022) Structures of *Tetrahymena's* respiratory chain reveal the diversity of eukaryotic core metabolism. Science 376, 831–839 10.1126/science.abn7747 35357889 PMC9169680

[56] Kao, M.C., Di Bernardo, S., Nakamaru-Ogiso, E., Miyoshi, H., Matsuno-Yagi, A. and Yagi, T. (2005) Characterization of the membrane domain subunit NuoJ (ND6) of the NADH-quinone oxidoreductase from *Escherichia coli* by chromosomal DNA manipulation. Biochemistry 44, 3562–3571 10.1021/bi0476477 15736965

[57] Kodera, N. and Ando, T. (2022) Visualization of intrinsically disordered proteins by high-speed atomic force microscopy. Curr. Opin. Struct. Biol. 72, 260–266 10.1016/j.sbi.2021.11.014 34998124

[58] Pal, N. (2022) Single-molecule FRET: a tool to characterize DNA nanostructures. Front. Mol. Biosci. 9, 835617 10.3389/fmolb.2022.835617 35330798 PMC8940195

[59] Kaledhonkar, S., Fu, Z., White, H. and Frank, J. (2018) Time-resolved cryo-electron microscopy using a microfluidic chip. Methods Mol. Biol. 1764, 59–71 10.1007/978-1-4939-7759-8_4 29605908

[60] Torino, S., Dhurandhar, M., Stroobants, A., Claessens, R. and Efremov, R.G. (2022) Time-resolved cryo-EM using a combination of droplet microfluidics with on-demand jetting. bioRxiv 2022.10.21.513149 10.1101/2022.10.21.513149 37592181

[61] Bongiovanni, G., Harder, O.F., Drabbels, M. and Lorenz, U.J. (2022) Microsecond melting and revitrification of cryo samples with a correlative light-electron microscopy approach. Front. Mol. Biosci. 9, 1044509 10.3389/fmolb.2022.1044509 36438663 PMC9685559

